# *CALB1* expression in meningioma: associations with progression-free survival and chromosome arm 6q loss

**DOI:** 10.1007/s00401-026-03113-3

**Published:** 2026-11-05

**Authors:** Lukas S. Friedrich, Friederike Liesche-Starnecker, Philipp Sievers, Claire Ertelt-Delbridge, Katharina J. Weber, Daniel Schrimpf, Nicole Pfarr, Patrick N. Harter, Jürgen Schlegel, Michael Meyer

**Affiliations:** 1 Institute of Physiology, Biomedical Center, LMU Munich, 80336 Munich, Germany; 2 Department of Neuropathology, Institute of Pathology, University Hospital Heidelberg, 69120 Heidelberg, Germany; 3 Clinical Cooperation Unit Neuropathology, German Cancer Research Center (DKFZ), German Consortium for Translational Cancer Research (DKTK), 69120 Heidelberg, Germany; 4 Institute of Neuropathology, Faculty of Medicine, Ulm University Center, Ulm University, 89081 Ulm, Germany; 5 Department of Neuropathology, Institute of Pathology, TUM School of Medicine and Health, Technical University of Munich, 81675 Munich, Germany; 6 Goethe University Frankfurt, University Hospital, Neurological Institute (Edinger Institute), 60528 Frankfurt Am Main, Germany; 7 Frankfurt Cancer Institute (FCI), 60596 Frankfurt Am Main, Germany; 8 Goethe University Frankfurt, University Hospital, University Cancer Center Frankfurt (UCT), 60590 Frankfurt Am Main, Germany; 9 German Consortium for Translational Cancer Research (DKTK) and German Cancer Research Center (DKFZ), Partner Site Frankfurt/Main, 60590 Frankfurt am Main, Germany; 10 Institute of Pathology, TUM School of Medicine and Health, Technical University of Munich, 81675 Munich, Germany; 11 German Cancer Research Center (DKFZ), German Consortium for Translational Cancer Research (DKTK), Partner Site Munich, 81377 Munich, Germany; 12 Institute of Neuropathology, LMU Medizin, LMU Munich, 81377 Munich, Germany; 13 Bavarian Cancer Research Center (BZKF), 81377 Munich, Germany; 14 Department of Neuropathology, Medical Faculty, Institute of Pathology, University of Augsburg, 86156 Augsburg, Germany

**Keywords:** Calbindin, *CALB1*, Calretinin, Meningioma, 6q loss

## Abstract

Molecular profiling has refined risk stratification in meningioma, but the biological relevance of associated transcriptional changes remains incompletely understood. Calbindin D28K (Calb), encoded by *CALB1*, is a calcium-binding protein with reported anti-apoptotic and cytoprotective functions. Previous unsupervised transcriptomic analysis identified *CALB1* upregulation in meningiomas with aggressive cellular features, but its biological and clinical relevance remains unresolved. We analysed Calb immunoreactivity in 231 meningioma tissue microarray (TMA) cores from 147 patients and evaluated *CALB1* transcript expression, DNA methylation and copy-number alterations in an independent cohort of 110 primary meningiomas. Calb immunoreactivity was detected in approximately 30% of primary tumours, and elevated *CALB1* transcript levels were observed at a similar frequency in the molecular cohort. High Calb protein expression was associated with shorter progression-free survival (PFS) after adjustment for the included clinical variables in the primary cohort. This association was also observed in restricted analyses of histological WHO grade 2 meningiomas. Lower methylation of selected exonic and intronic *CALB1* CpG sites correlated with a higher transcript expression, consistent with an epigenetic contribution to deregulation. Multivariable analyses identified associations of *CALB1* transcript expression with chromosome arm 6q loss, histological WHO grade, *CALB1* hypo-methylation and specific methylation subclasses in the secondary cohort. Differential expression analysis of tumours harbouring 6q loss identified expression differences in *CALB1* and transcription factors, including *NKX2-5* and *SOX11*. In contrast, expression of a related calcium-binding protein Calretinin (Calr) and the Progesterone Receptor (PR) was not significantly associated with PFS in the primary cohort. Together, these findings support the potential prognostic relevance of Calb protein expression, particularly in histological WHO grade 2 meningiomas, and associate *CALB1* transcript expression with selected molecular features in complementary cohorts. Calb immunohistochemistry may therefore provide a practical tool for refining risk stratification in this clinically heterogeneous tumour type prior to integrative molecular testing.

**Supplementary Information:**

The online version contains supplementary material available at https://doi.org/10.1007/s00401-026-03113-3.

## Introduction

Meningiomas are the most common primary intracranial neoplasms and arise from arachnoid cap cells of the leptomeninges. They comprise a biologically heterogeneous group of tumours characterised by diverse histological subtypes and molecular alterations. Whilst most meningiomas follow a relatively favourable clinical course, the current *World Health Organization Classification of Tumors of the Central Nervous System* (CNS WHO) [23] recognises a spectrum ranging from indolent WHO grade 1 lesions to highly aggressive WHO grade 3 tumours. In addition to classical histopathological criteria—including mitotic activity, brain invasion and specific morphological features—molecular profiling has substantially refined prognostic stratification. Chromosomal copy-number alterations have emerged as key determinants of tumour progression. By this rationale, cIMPACT-NOW update 8 recommends assigning CNS WHO grade 2 to meningiomas with grade 1 morphology that harbour chromosome arm 1p deletion together with 22q deletion and/or an oncogenic *NF2* variant [29]. These recommendations extend molecular risk assessment beyond the criteria of the 2021 CNS WHO classification [23]. Losses of 6q and 14q have also been frequently associated with adverse clinical outcomes [24]. In addition, DNA methylation-based classification schemes provide further prognostic resolution when integrated with WHO grade and chromosomal aberrations, as exemplified by the integrated molecular score (IMS) [24]. Despite these advances, the downstream molecular programmes associated with aggressive behaviour of meningiomas harbouring these chromosomal aberrations remain incompletely understood.

Calb is a member of the EF-hand calcium-binding protein family with established buffering and sensor-like functions in calcium homeostasis [30]. In humans, its expression is largely restricted to cerebellar Purkinje neurons [9], selected neuronal subpopulations [9] and epithelial cells of the distal nephron and collecting duct [6]. Beyond its physiological role, increasing experimental evidence suggests tumour-promoting properties. Calb has been shown to inhibit caspase-3-mediated apoptosis [5], facilitate escape from cellular senescence [1] and influence proliferation [15]. Consistent with these findings, Calb expression has been reported in several epithelial [1, 7, 16] and CNS neoplastic entities [21, 25, 34]. In meningioma, however, data on Calb remain scarce and contradictory. *CALB1* was previously identified in unbiased transcriptomic analyses of meningioma as part of a stemness-associated gene signature linked to aggressive and drug-resistant tumour phenotypes [18]. Corresponding primary meningioma cell lines with aggressive growth characteristics also exhibited lower nuclear caspase-3 immunoreactivity [18]. However, early immunohistochemical analyses in a small posterior fossa meningioma cohort did not detect Calb immunoreactivity [17]. Thus, the presence, regulatory mechanisms, and clinical relevance of Calb in meningioma remain unresolved.

Given its anti-apoptotic potential and the incomplete understanding of transcriptional changes associated with molecularly defined high-risk meningiomas, we hypothesised that *CALB1* expression is associated with clinical outcome and adverse molecular features. We investigated Calb protein expression and its clinicopathological and prognostic associations in a primary immunohistochemical cohort. In an independent secondary cohort, *CALB1* transcript expression, DNA methylation and recurrent copy-number alterations were comprehensively analysed. These complementary analyses aimed to characterise *CALB1*-associated biology and evaluate its potential prognostic relevance.

## Materials and methods

### Human tissues

To analyse the expression of Calb in meningioma and compare it to clinical data, a primary cohort of 231 TMA cores, corresponding to 179 samples of 147 patients, was assembled. This extensive and already comprehensively clinically documented cohort of predominantly high-grade meningiomas was selected for a focussed evaluation of the association between clinicopathological parameters, Calb expression in the tumours, and recurrence during follow-up. The samples were provided by the UCT Biobank Frankfurt. All clinical and follow-up data are depicted in Table 1. Whilst no molecular data were available, samples were graded according to the 2016 version of the CNS WHO classification [22], which was the valid version at the time of study initiation. In the primary cohort, analyses were restricted to primary meningiomas, except for the univariable and multivariable Cox and linear regression analyses, which additionally included recurrent tumours. In addition, to compare meningiomas with their tissue of origin, 13 completely and irreversibly anonymised meningeal samples from routine autopsies, conducted at the Department of Neuropathology, Institute of Pathology, Technical University of Munich, were included as reference.

**Table 1:** Clinical data of the primary cohort

Median age (range, years)	58 (17—84)
Sex, *n* (%)	
Female	82 (55.8)
Male	60 (40.8)
N/A	5 (3.4)
Primary/Recurrences, *n* (%)	
Primary	133 (90.5)
Recurrence	14 (9.5)
WHO grade (2016) *n* (%)	
I	1 (0.7)
II	135 (91.8)
III	11 (7.5)
Subtypes, *n* (%)	
Atypical	124 (84.4)
Clear cell	1 (0.7)
Chordoid	10 (6.8)
Anaplastic	11 (7.5)
N/A	1 (0.7)
Side, *n* (%)	
Left	68 (46.3)
Right	60 (40.8)
Both	9 (6.1)
Other	10 (6.8)
Simpson grade, *n* (%)	
I	53 (36.1)
II	42 (28.6)
III	15 (10.2)
IV	32 (21.8)
N/A	5 (3.4)
Median MIB-1% (range)	6 (3—40)
Location, *n* (%)	
Frontal	55 (37.4)
Central	15 (10.2)
Sphenoid wing	13 (8.8)
Parietal	11 (7.5)
Fronto-parietal	7 (4.8)
Other	46 (31.3)
Median F/U recurrence (range, months)	28 (0—237)
Recurrences F/U, *n* (%)	45 (30.6)
Median F/U survival (range, months)	35 (0—186)
Death F/U, *n* (%)	15 (10.2)

A secondary cohort comprising 110 publicly available primary meningioma datasets with associated clinical information was included to enable molecular comparisons across histological WHO grades 1 and 2. All cases were originally classified according to the 2016 version of the CNS WHO classification [22]. Using available copy-number profiles and *NF2* as well as *TERT* promoter mutation status (*pTERT*), we applied a modified integrated grading scheme based on the 2021 CNS WHO framework [23] and the cIMPACT-NOW update 8 recommendations [29]. Informed by recent findings on the context-dependent prognostic relevance of *pTERT* mutations [10], *pTERT*-mutant tumours were upgraded to grade 3 only when their original histological grade was 2. Tumors with grade 1 morphology were not upgraded solely on the basis of a *pTERT* mutation. The modified integrated grading scheme was used for the secondary-cohort survival analyses and the WHO grade expression comparisons, whereas original histological WHO grades were retained for the clinical–molecular regression analyses. Clinical characteristics and follow-up data are summarised in Table 2.

**Table 2:** Clinical data of the secondary cohort

Median age (range, years)	59.5 (19—81)
Sex, *n* (%)	
Female	67 (60.9)
Male	43 (39.1)
Primary/Recurrences, *n* (%)	
Primary	110 (100.0)
Recurrence	0 (0.0)
Original WHO grade (2016), *n* (%)	
I	90 (81.8)
II	20 (18.2)
WHO grade (2021), *n* (%)	
1	66 (60.0)
2	7 (6.4)
3	11 (10.0)
N/A	26 (23.6)
Modified integrated WHO grade (cIMPACT-NOW 8 / *pTERT*), *n* (%)	
1	72 (65.5)
2	25 (22.7)
3	3 (2.7)
N/A	10 (9.1)
Simpson grade, *n* (%)	
I	37 (33.6)
II	48 (43.6)
III	0 (0.0)
IV	24 (21.8)
N/A	1 (0.9)
Median MIB-1% (range)	3.6 (0.5—32.7)
Location *n* (%)	
Sphenoid wing	20 (18.2)
Parafalcine	17 (15.5)
Frontal	16 (14.5)
Anterior skull base	16 (14.5)
Petroclival	10 (9.1)
Other	31 (28.2)
Median F/U (range, months)	23.2 (0—96.5)
Recurrences F/U, *n* (%)	19 (17.3)

The two cohorts were complementary. The primary cohort was used to investigate Calb protein expression and its clinical associations. In the secondary cohort, *CALB1* transcript expression, DNA methylation and CNA status were analysed in the same tumours. Calb protein expression was not available for these molecularly characterised tumours.

### Immunohistochemistry

5 µm-thick, ready-to-use sections of formalin-fixed, paraffin-embedded (FFPE) TMA samples were deparaffinised and rehydrated, using a series of xylene, ethanol dilutions and DM water, followed by antigen retrieval in pH 6.0 citrate buffer (95 °C) and quenching of endogenous peroxidase with 1.5% hydrogen peroxide solution. Nonspecific antibody binding sites were blocked, using blocking buffer with 2.5% normal horse serum (Vector Laboratories, USA). Incubation with primary antibody solutions, containing avidin (Vector Laboratories, USA), was performed overnight at 4 °C, with subsequent binding of secondary antibodies and masking of avidin with biotin (Vector Laboratories, USA) for 30 min. All primary and secondary antibodies are listed in Table 3. Visualisation of antigen–antibody complexes was done by incubation with ABC-reagent (Vector Laboratories, USA), containing horseradish peroxidase (HRP) for 30 min, followed by 3,3´-diaminobenzidine (DAB, Vector Laboratories, USA). After counterstaining with haematoxylin for 5 min and blueing, slides were dehydrated using ethanol dilutions and xylene and finally mounted.

**Table 3:** Antibody properties used for immunohistochemistry

Antibody	Usage	Host species	Product number	Clonality	Final dilution	Manufacturer; RRID
Anti-calbindin	Primary	Mouse	C9848	Monoclonal	1:500	Sigma-Aldrich, Inc. (St. Louis, USA); AB_476894
Anti-calbindin	Primary	Rabbit	CB38a	Polyclonal	1:5000	Swant AG (Burgdorf, Switzerland); AB_3107026
Anti-calretinin	Primary	Rabbit	CR7697	Polyclonal	1:5000	Swant AG (Burgdorf, Switzerland); AB_2619710
Anti-progesterone Receptor	Primary	Mouse	Clone 16 (PR16)	Monoclonal	1:100	DCS, Dr. Christian Sartori GmbH & Co. KG (Hamburg, Germany)
Anti-rabbit IgG Antibody (H+L) Biotinylated	Secondary	Horse	BA-1100	IgG	1:400	Vector Laboratories, Inc. (Newark, United States); AB_2336201
Anti-mouse IgG Antibody (H+L) Biotinylated	Secondary	Horse	BA-2000	IgG	1:400	Vector Laboratories, Inc. (Newark, United States); AB_2313581

Final concentrations of all antibodies were determined by serial dilutions prior to the start of experiments (Table 3). Slides of cerebellum (Calb, Calr) and breast cancer metastasis (PR) were used as positive controls in each run. Negative controls were prepared by omitting the primary antibody.

### Digitisation and evaluation

All stained slides were scanned using an Aperio slide scanner (Aperio AT2, Leica Biosystems, Germany) and converted to.svs files. Calb and PR status were assessed using a semi-automated, quantitative cell detection and scoring-algorithm implemented in the open-source software QuPath 0.5.0, as previously described for PR status [4]. Following background correction and colour deconvolution to separate Haematoxylin and DAB channels, tumour cell nuclei were identified using the *Positive Cell Detection* command with customised parameter settings. Tumour cell nuclei identification was based on optical density within the Haematoxylin channel. The H-scores were calculated by applying three intensity thresholds for cellular (Calb) or nuclear (PR) DAB intensity. Afterwards, multiple regions of interest (ROIs) from several Calb- and PR-stained TMA slides were selected to train a cell classifier to exclude detections corresponding to faulty results from erythrocytes, haematoxylin crystals and other artefacts. After application of the complete workflow to all TMA samples, an individual grading system based on H-score calculation was applied for Calb (negative: <10, weak positive: ≥10 to <50, intermediate positive: ≥50 to <100, strong positive: ≥100) and for PR (negative: <10, weak positive: ≥10 to <100, strong positive: ≥100). In tumour cases with multiple cores, the mean H-score was calculated. Calr immunohistochemistry, however, was manually evaluated in categories due to its generally weak staining results (negative: no positive tumour cells, positive: singular or singular groups of positive tumour cells).

### RNA sequencing data

Raw count matrices from two publicly available meningioma RNA sequencing (RNA-seq) datasets (GSE189672 [2] and GSE139651 [26]) were obtained from the *Gene Expression Omnibus* (GEO) database of the *National Center for Biotechnology Information* (NCBI) [8], together with the corresponding metadata. RNA-seq data were processed in R (version 4.2.1) using edgeR (version 3.38.4) and limma (version 3.52.4) packages. Raw counts were restricted to genes present in both datasets, filtered for lowly expressed genes and normalised for library size using the trimmed mean of M-values (TMM) method. Log2 CPM values were batch-corrected using limma’s *removeBatchEffect()* function whilst preserving tumour versus arachnoid reference status. Corrected *CALB1* expression values were used for visualisation and expression-category assignment. Data from 110 primary meningioma samples (GSE189672 [2]) were then used for the secondary cohort, whereas 2 arachnoid granulation cell model samples (GSE139651 [26]) served as reference for *CALB1* expression levels. For regression analyses, TMM-normalised log2 CPM values were generated separately from all 110 secondary-cohort tumours, without prior batch correction; sequencing batch was included directly as a model covariate.

### Methylation data

To assess global DNA methylation patterns and CpG sites within the *CALB1* locus, publicly available methylation BeadChip samples from the NCBI GEO repository GSE189521 [2] were used.

To compare *CALB1* methylation between meningiomas and tissues with intrinsically either a high or low Calb expression, .idat files from 13 normal lung samples (GSE173356 [20]) as well as seven normal cerebellum and seven normal cerebrum samples (GSE154403 [11]) were incorporated into the analysis.

Two different array versions were used within the data processing (Illumina EPIC and 450 K methylation array). Common probes were identified according to the manufacturer’s annotations, allowing both array types to be merged using the minfi package (version 1.43.1) in R (version 4.2.2). For comparison of different samples of several batches, a background and dye-bias correction, followed by log2-transformation of the values was performed. In the next step, batch effects related to array type and tissue preservation were addressed using the limma package, with methylated and unmethylated signals corrected separately. Beta values were subsequently calculated according to

$$\beta = \frac{M}{M+U+ \alpha }$$

where M and U denote the re-transformed methylated and unmethylated signal intensities, and *α* represents an offset of 100. Copy-number variation plots and methylation-based classifier scores of the Heidelberg Brain Tumor Classifier V12.8 were generated as previously described [32].

Chromosomal copy-number alterations were inferred from Illumina Infinium MethylationEPIC v1.0 BeadChip data using the conumee R package as previously described [13]. All secondary-cohort tumour samples were profiled on this platform. Raw.idat files were imported using minfi and processed with *preprocessRaw*, without background correction, dye-bias correction or control-probe normalisation at this preprocessing stage. Query samples were subsequently normalised against a linear combination of 50 sex-matched copy-number-neutral control samples. Log2 ratios between observed and fitted intensities were aggregated into 26,446 genomic bins (GRCh37/hg19), baseline-centred using conumee and segmented by circular binary segmentation with alpha = 0.001, 50,000 permutations, a minimum of five bins per segment, undo.splits = "sdundo" and undo.SD = 2.2. Thresholds were applied to individual segments: segments with a mean log2 ratio ≥ +0.10 were called gains, and those with a mean log2 ratio ≤ −0.10 were called losses. Chromosomal arms were categorised according to the fraction of their segment-covered length affected by copy-number alterations. Segments spanning a centromere were split at the p/q boundary, and the resulting portions were assigned to the respective arms. For each alteration direction, changes affecting ≥5% and ≤25% of the assessed arm length were classified as focal, changes affecting >25% and ≤95% as segmental, and changes affecting >95% as complete arm-level gains or losses. Arms harbouring both gains and losses, each affecting ≥5% of the assessed arm length, were classified as gain/loss. Arms with <5% altered genomic content were considered balanced. Arm-level extent categories were adapted from the framework described by Hielscher et al. [13], with an additional distinction between focal and larger segmental alterations for descriptive reporting. For linear expression regression analyses, segmental and complete arm-level alterations were merged into single gain or loss categories, respectively. Focal alterations and gain/loss classifications were coded as missing owing to their low frequency and ambiguous interpretation. To reduce model complexity, *p*- and *q*-arm CNA calls for chromosomes 10 and 18 were combined into composite chromosome-level categories. When one arm was balanced, focal or segmental alterations of the other arm retained their category, whereas complete arm-level gains or losses were classified as segmental chromosome-level gains or losses, respectively. Concordant arm-level categories were retained, same-direction combinations of segmental and complete alterations were classified as segmental, and opposite-direction alterations were classified as gain/loss.

### Statistical analysis

Continuous clinicopathological parameters were compared between expression groups using two-sided Mann–Whitney U tests. The same test was used for Calb and PR H-scores across clinicopathological groups in the primary cohort and for CpG beta values across tissue/expression groups in the secondary cohort. Pairwise comparisons were Holm-adjusted within each factor or CpG site.

Linear regression analyses used heteroscedasticity-consistent HC1 robust standard errors and model-specific complete cases. In the primary cohort, the outcome was log2-transformed Calb H-score; the multivariable model included age, sex, primary versus recurrent disease status, and histological grade/subtype. In the primary cohort, the single histological WHO grade 1 tumour and the single clear cell meningioma were excluded from linear regression models including histological grade/subtype because each category contained only one case. Secondary-cohort regression analyses used TMM-normalised log2 CPM generated from all 110 tumours, without prior batch correction, and included sequencing batch as a covariate. Batch-corrected values were used only for visualisation and expression-category assignment. Single-factor models included one variable of interest and batch. The clinical–molecular multivariable model included batch, 1p, 6q and 22q status, original histological WHO grade, age, sex, *TERT* promoter status, methylation subclass, and the mean beta value of cg26290632, cg12402251 and cg18335796. The single *CDKN2A/B* homozygously deleted tumour was excluded from these clinical–molecular models but retained for normalisation and other analyses. A chromosome-focussed model included batch and CNA categories for 1p, 1q, 6q, chromosome 10, 14q, chromosome 18 and 22q. Its targeted 6q-loss versus balanced contrast used a nominal p value; other CNA coefficients were reported descriptively.

Secondary-cohort pairwise contrasts were calculated using emmeans and Holm-adjusted within each factor. Mutation-status comparisons were jointly Holm-adjusted across *NF2*, *SMARCB1*, *TRAF7*, *KLF4*, *AKT1*, *PIK3CA*, *SMO*, *POLR2A* and *TERT* promoter status. Models restricted to a single batch omitted the batch term. *ARID1A* was displayed descriptively and excluded from inference because only one mutant tumour occurred within the batch containing both status groups.

Exploratory coefficient tests were Benjamini–Hochberg FDR-adjusted separately within the primary-cohort univariable and multivariable analyses, secondary-cohort single-factor and multivariable clinical–molecular analyses, and single-factor CNA analyses. Intercept and batch coefficients were excluded. Additional batch-adjusted models examined CNA-*ESR1*/*ESR2* and *ESR*-*CALB1* associations. Rank-based sensitivity analyses ranked both *CALB1* and the *ESR* predictor. FDR correction was applied separately across the CNA-*ESR* coefficient tests, two linear *ESR*-*CALB1* tests, and two rank-based tests. Linear-model estimates, confidence intervals, reference categories and sample sizes are provided in Supplementary File 1.

PFS was analysed using Kaplan–Meier estimates, log-rank tests, and univariable and multivariable Cox proportional hazards models. The primary multivariable model included age, sex, Calb expression group, WHO grade, extent of resection, and primary versus recurrent disease status. Radiotherapy was evaluated separately as a time-dependent covariate in a univariable model. The restricted secondary model included age, sex, *CALB1* expression group, extent of resection, and modified integrated WHO grade. Cox models used model-specific complete cases. Hazard ratios are reported with 95% confidence intervals and nominal two-sided Wald *p* values.

DESeq2 analyses used raw RNA sequencing counts and included sequencing batch. Tumors with segmental or whole-arm 6q loss were compared with all remaining eligible tumours and, separately, within the 1p/22q-codeleted subgroup. Focal or mixed alterations precluding group assignment were excluded. Wald-test *p* values were Benjamini–Hochberg-adjusted within each genome-wide comparison. apeglm-shrunken log2 fold changes [38] were used for visualisation and ranking; volcano plots highlighted adjusted *p*<0.01 and absolute shrunken log2 fold change >2. Differential expression analyses used R 4.2.1 and DESeq2 1.36.0. Other statistical analyses used R 4.5.0 with ggplot2 4.0.3, survminer 0.5.0, broom, dplyr, lmtest, sandwich, emmeans, edgeR, limma, and survival. Nominal or adjusted *p*<0.05 defined statistical significance, as applicable. Final layouts were edited using Inkscape 1.4.2.

## Results

### Patient data

**Fig. 1:**
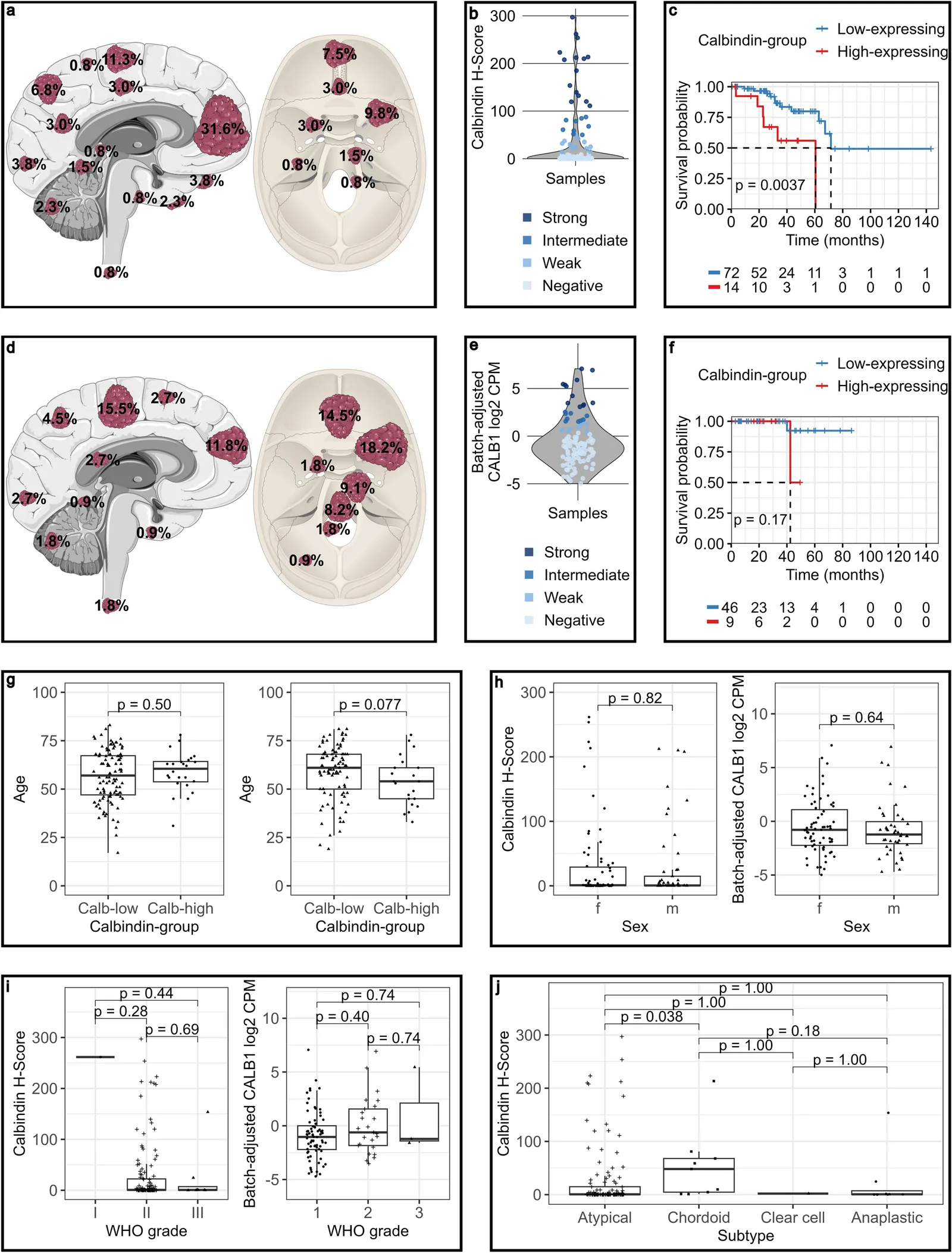
Clinicopathological parameters of the primary and secondary cohort. **a**, **d** Distribution of the most common anatomical locations amongst primary meningiomas in the primary (a) and secondary (d) cohort, shown as relative frequencies. Calb H-scores in the primary (b) and corresponding batch-adjusted CALB1 log2 CPM in the secondary cohort (e) are shown, with stratification into the expression categories “negative”, “weak”, “intermediate” and “strong”. **c**, **f** PFS of patients with “high” versus “low” Calb-expressing primary, gross-totally resected meningiomas in the primary (c) and secondary (f) cohort. Survival analyses are restricted to the WHO grade with the highest case number in each cohort (non-irradiated WHO grade II meningiomas in the primary cohort; WHO grade 1 meningiomas in the secondary cohort). **g–j** Comparison of selected clinicopathological variables according to Calb-expression status of primary tumours. *Image credits*: Panels (a) and (d) each contain elements adapted from three illustrations provided by both Servier Medical Art (https://smart.servier.com) and Human Bio Media (https://humanbiomedia.org). All source illustrations are licenced under the Creative Commons Attribution 4.0 International License (CC BY 4.0, https://creativecommons.org/licenses/by/4.0/)

The primary cohort comprised 147 patients, including 133 primary meningiomas and 14 non-corresponding recurrent tumours. All cases were reviewed by experienced neuropathologists and classified according to the 2016 CNS WHO version [22]. The secondary cohort consisted of 110 publicly available primary meningiomas, originally diagnosed according to the 2016 WHO criteria [22]. *TERT* promoter mutation status was unavailable for 26 tumours. Under the modified integrated grading scheme described in *Methods*, missing *pTERT* status did not preclude classification of tumours with original histological WHO grade 1. Molecular grading was therefore possible for 100 tumours. This resulted in 72 tumours (72.0%) classified as WHO grade 1, 25 tumours (25.0%) as WHO grade 2, and 3 tumours (3.0%) as WHO grade 3. Pathogenic *pTERT* mutations (C228T or C250T) were identified in 9.1% of the cases, including 2 tumours previously classified as WHO grade II. Homozygous *CDKN2A/B* deletion was detected in 1 tumour (previously WHO grade II). Detailed clinicopathological characteristics of both cohorts are summarised in Tables 1 and 2, common anatomical locations are depicted in Fig. 1a, d.

### Calbindin expression

Antibody specificity was confirmed using cerebellar Purkinje cells as positive and omission of the primary antibody as negative controls (Supplementary Fig. 1a–d). After verification of tumour cell content >90% on H&E staining, 132 primary meningiomas from the primary cohort were evaluable; one case was excluded for technical reasons. Using a modified H-score system, Calb immunoreactivity was detected in 28.8% of tumours (Fig. 1b). The distribution of staining intensities is summarised in Table 4.

**Table 4:** Results of Calb-IHC staining of primary meningiomas of the primary cohort

Result	H-score	*n* (%)
Negative	0—<10	94 (71.2)
Weak positive	≥10—<50	14 (10.6)
Intermediate positive	≥50—<100	9 (6.8)
Strong positive	≥100	15 (11.4)

**Fig. 2:**
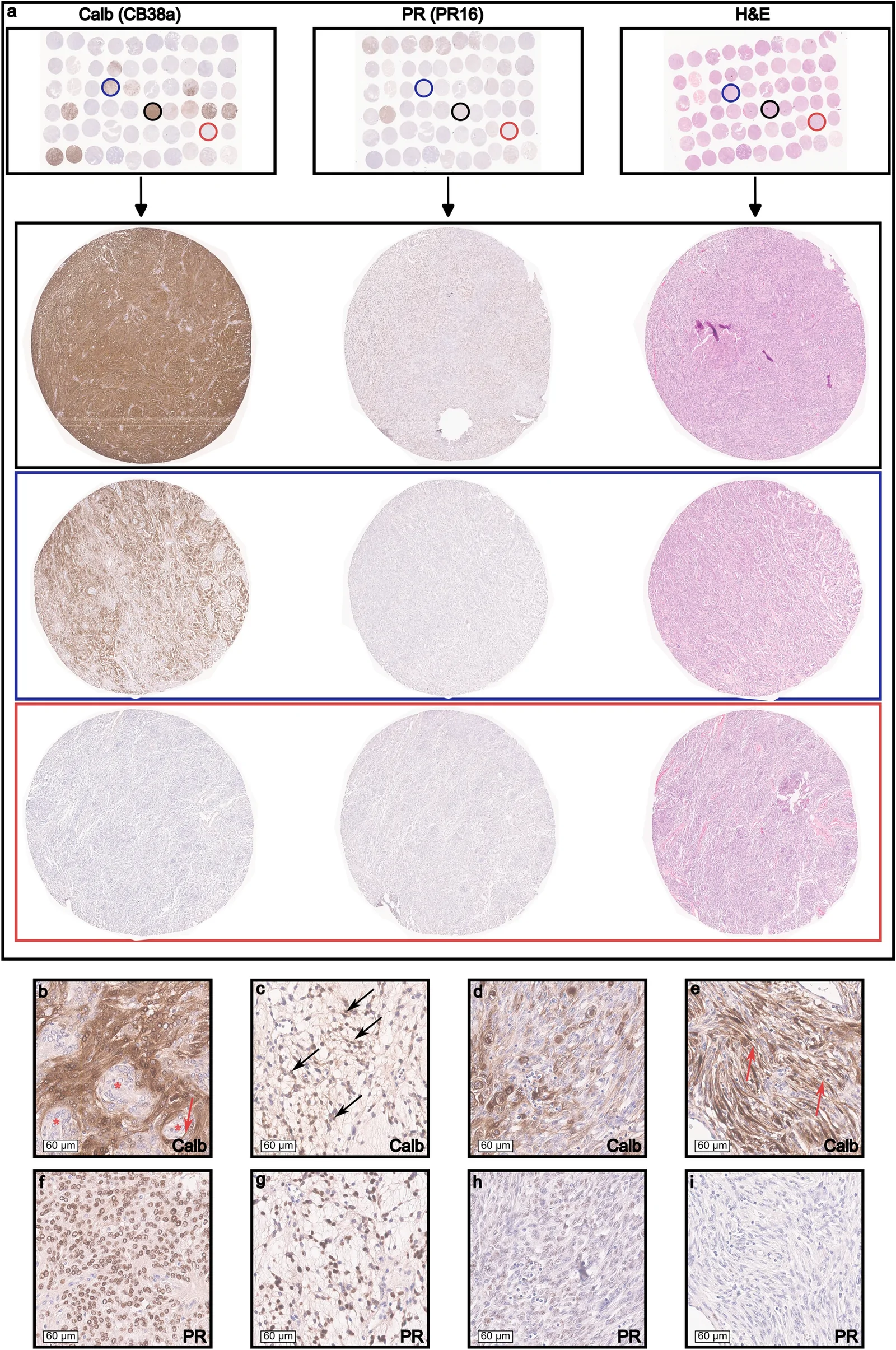
Immunohistochemical analysis of Calb and PR expression. **a** Representative immunostainings of TMA cores from the primary cohort. Upper row (black): atypical meningioma (WHO grade II) with strong Calb expression and intermediate nuclear PR staining. Middle row (blue): anaplastic meningioma (WHO grade III) showing moderate Calb expression without detectable nuclear PR immunoreactivity. Lower row (red): atypical meningioma (WHO grade II) lacking both Calb and PR expression. **b–e** Representative immunostainings illustrating Calb distribution with a typical diffuse staining pattern in (b) and (e), as well as areas of negative tumour cells (b–d) in otherwise intermediate to strong Calb-expressing samples. Black arrows indicate tumour cells with predominantly nuclear Calb staining in a chordoid meningioma. Red arrows highlight membranous Calb staining in isolated tumour cells. Red asterisks mark clusters of Calb-negative tumour cells within otherwise positive tumour regions. **f–i** Representative PR immunostainings demonstrating strong (f, g), weak (h), and absent (i) nuclear immunoreactivity

Intratumoural Calb immunoreactivity was predominantly observed in both the cytoplasm and nucleus, most commonly showing a diffuse cellular staining pattern. Rarely immunoreactivity was restricted exclusively to the nucleus or the cytoplasm without nuclear labelling (Fig. 2b–e). Tumors with intermediate to strong Calb expression generally exhibited diffuse staining throughout large tumour areas, with only occasional small, interspersed clusters of negative cells. In contrast, weak-to-intermediate positive meningiomas showed predominantly focal staining limited to clusters of tumour cells, whereas negative cases contained a few scattered positive cells at most. Normal meningeal cells did not show any immunoreactivity in 9 of 13 cases as demonstrated in Supplementary Fig. 1e. In the remaining 4 samples, the vast majority of meningeal cells was negative, with a small number of weak positive cells, predominantly located in the basal layers of the arachnoid mater (Supplementary Fig. 1f). For downstream analyses, “negative” and “weak positive” samples were combined into a Calb-low group (81.8%), whereas “intermediate positive” and “strong positive” samples defined the Calb-high group (18.2%). In the independent secondary cohort, positivity was defined as ≥2.5-fold to <5.5-fold relative to the mean *CALB1* expression of the control arachnoid granulation cell samples, with a high Calb-expression determined as ≥5.5x-fold (intermediate: ≥5.5-fold to <11.5-fold; strong: ≥11.5-fold, respectively). Using these thresholds, 8 of 110 cases (7.3%) showed weak *CALB1* expression, whereas 21 cases (19.1%) demonstrated intermediate or strong *CALB1* transcript levels, closely mirroring the distribution observed at the protein level (Fig. 1e).

Systematic evaluation of clinicopathological parameters of the primary cases revealed no significant association between Calb expression and patient sex in either cohort in univariable analysis, with positive tumours detected in 30.7% and 28.4% of female and 26.4% and 23.3% of male patients, respectively (Fig. 1h). Likewise, median age did not differ significantly between patients with Calb-high (60.5/54 years) and Calb-low tumours (57/61 years; Fig. 1g). No preferential neuroanatomical localisation of Calb-high tumours was observed (Supplementary Fig. 2f). Mitotic activity and proliferative index failed to discriminate between Calb-low and -high tumours, with comparable proportions of Calb-positive cases amongst tumours with low (30%), intermediate (28.7%), and high (25%) mitotic counts (Supplementary Fig. 2a). Accordingly, median Ki-67 indices were similar between Calb-low and -high tumours (6% vs. 5% / 3.65% vs. 3.5%; Supplementary Fig. 2e). Comparison across WHO grades in the primary cohort revealed no significant differences as shown in Fig. 1i. In the secondary cohort, histological WHO grade 2 was associated with a higher *CALB1* transcript expression after adjustment for the covariates in the clinical–molecular model (*p*=0.023; Supplementary File 1). In the primary cohort, chordoid tumours showed higher Calb H-scores than atypical tumours in pairwise analysis (*p*=0.038; Fig. 1j). This association was also observed in the multivariable model (*p*=0.015; Supplementary File 1).

**Fig. 3:**
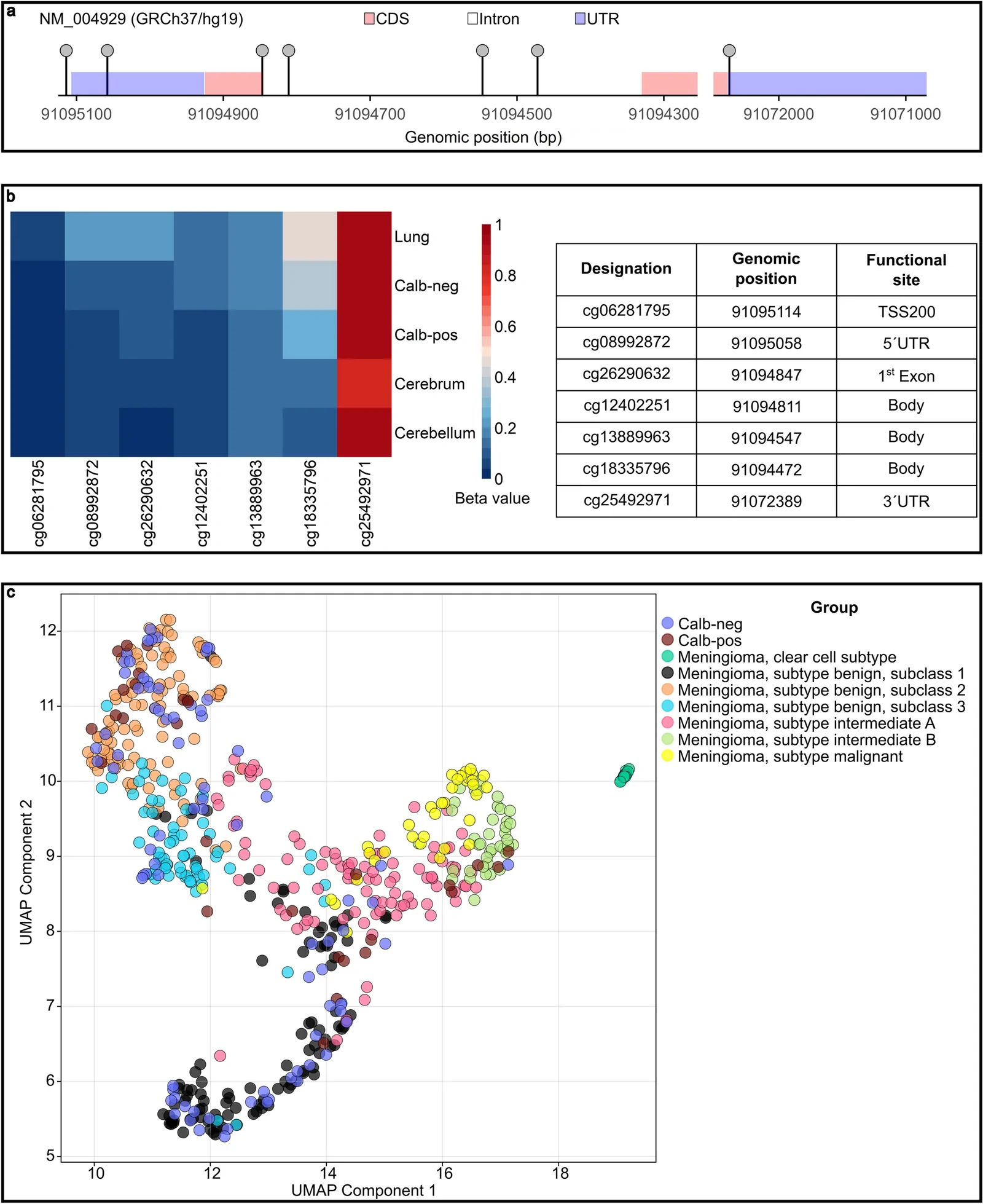
Methylation analysis of CALB1 CpG sites in the secondary cohort. **a** All seven evaluable CALB1 CpG sites and their location. **b** A heatmap of mean beta values across all CALB1 CpG sites in the investigated tissues reveals progressive hypomethylation at exonic and intronic CALB1 CpG sites with increasing CALB1 expression, in contrast to the predominantly hypermethylated pattern observed in lung tissue. Genomic positions and functional aspects of all CpG sites are depicted. **c** UMAP-based dimensionality reduction of 5000 CpG sites illustrating global methylation patterns across all meningiomas compared to reference meningioma samples

UMAP-based dimensionality reduction of the secondary cohort together with 453 meningioma samples from the Heidelberg Brain Tumor Classifier V12.8 reference set demonstrated a broad distribution of Calb-positive and -negative tumours without formation of a distinct methylation cluster (Fig. 3c and Supplementary Fig. 2c). Relative to methylation subclass “Benign 1”, “Benign 3” was associated with a higher CALB1 transcript expression (*p*<0.05), whereas “Intermediate B” was associated with a lower expression (*p*=0.013; Supplementary File 1) in the multivariable analysis. Calb expression was neither associated with TERT promoter mutation status nor IMS-category (Supplementary Fig. 2b, d). CALB1 expression was further analysed across the major molecular driver alterations associated with meningioma subgroups (NF2, SMARCB1, TRAF7, KLF4, AKT1, PIK3CA, SMO, POLR2A and ARID1A; Supplementary Fig. 3). Amongst evaluable driver-mutation comparisons, no association with CALB1 expression remained statistically significant after adjustment for multiple testing. Taken together, Calb expression showed only limited associations with conventional clinicopathological parameters, whereas multivariable analyses demonstrated independent associations with histologically defined WHO grade and distinct molecular features.

**Fig. 4:**
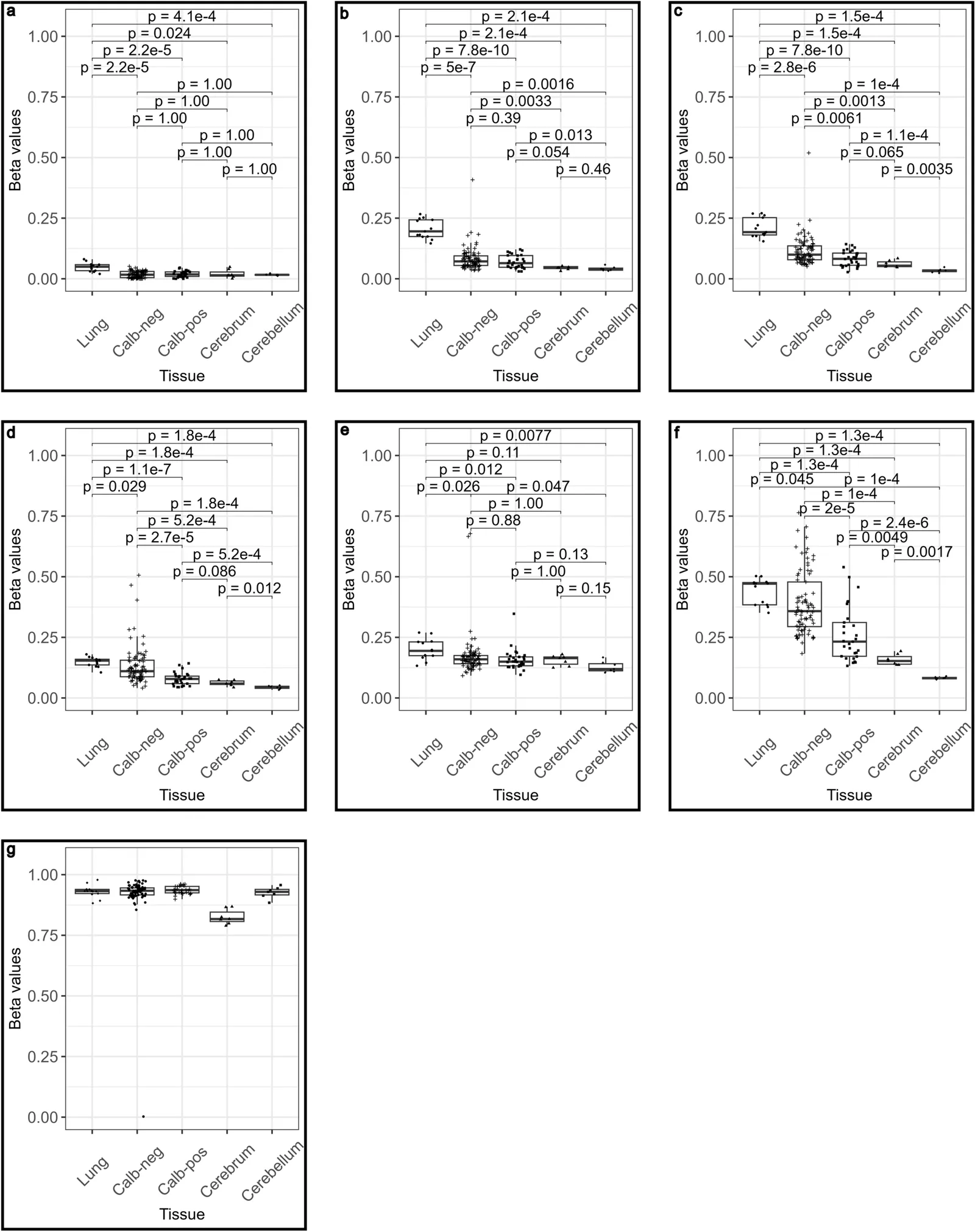
Methylation patterns of Calb-positive and -negative samples in the secondary cohort. **a–g** Comparison of beta values across the seven available CALB1-associated CpG sites demonstrates significant differences between Calb-negative and -positive meningiomas at cg26290632 (c), cg12402251 (d) and cg18335796 (f) (*p*=0.0061, *p*=2.7e−5, 2e−5, respectively). **a** cg06281795, **b** cg08992872, **c** cg26290632, (d) cg12402251, (e) cg13889963, **f** cg18335796, **g** cg25492971. In panel (g), Holm-adjusted *p* values were <0.01 for all comparisons involving cerebrum and 1.00 for all remaining comparisons

To date, little is known about the regulatory mechanisms controlling *CALB1*, especially in meningioma. To investigate potential epigenetic mechanisms underlying *CALB1* upregulation, DNA methylation patterns of the secondary cohort were analysed. Of 12 *CALB1*-associated CpG sites represented on the Illumina EPIC 850 K array, 7 probes were shared with the 450 K array and included in the analysis. Beta values were compared between positive and negative samples, using lung tissue (low intrinsic expression), cerebellar and cerebral cortex tissue (high intrinsic expression) as references. Probe designations, chromosomal positions and functional sites—according to the manufacturer’s annotation files—can be seen in Fig. 3b. Significant differences between positive and negative meningiomas were observed for three CpG sites (cg26290632, cg12402251, cg18335796; Fig. 4c, d, f), located within the 1st exon or intron of *CALB1*, with cg18335796 showing the strongest relative hypo-methylation (*p*=2e^−5^; Fig. 4f). The remaining four CpG sites (cg25492971, cg06281795, cg08992872 and cg13889963), located within the 3´UTR, promoter region, 5´UTR and 1st intron, exhibited only minor differences between all samples (Fig. 4a, b, e, g). Overall, meningiomas and normal CNS-tissue displayed relative hypo-methylation across most *CALB1* CpG sites compared with lung tissue (Fig. 3a, b). Lower values of the *CALB1* CpG scores (mean of the three sites cg26290632, cg12402251, cg18335796) were associated with a higher transcript expression in the clinical–molecular regression model (*p*=0.0084; Supplementary File 1).

As *CALB1* expression has previously been linked to oestrogen receptor signalling, *ESR1* and *ESR2* expression was analysed in relation to chromosome arm 6q and 14q status, as well as *CALB1* expression. *ESR2* expression was lower in tumours with 6q loss and 14q loss (*p*=1.94e−5 and *p*=0.019, respectively). *ESR1* expression was higher in tumours with 14q loss (*p*=0.029), whereas no association with 6q loss was observed. Neither *ESR1* nor *ESR2* transcript expression was significantly associated with *CALB1* transcript expression in the corresponding analyses (Supplementary File 1).

### Calretinin expression

Calr, a calcium-binding protein closely related to Calb, was also assessed via IHC. Calr protein expression was sparse and restricted to a small subset of tumours, involving both cytoplasmic and nuclear compartments. Twelve of 132 primary cases (9.1%) displayed isolated scattered, predominantly strong positive tumour cells (Supplementary Fig. 1 g), consistent with previous findings describing a low-level Calr expression in meningiomas [31]. Owing to the limited number and focal distribution of positive cells, no further clinicopathological variables were investigated.

### Progesterone receptor expression

To contextualise and compare Calb expression with an established immunohistochemical differentiation marker in meningioma, nuclear PR expression was assessed in all 133 primary meningiomas, resulting in 48.1% positive samples, as demonstrated in Table 5.

**Table 5:** Results of PR-IHC staining of primary meningiomas of the primary cohort

Results	H-score	*n* (%)
Negative	0—<10	69 (51.9)
Weak positive	≥10—<100	43 (32.3)
Strong positive	≥100	21 (15.8)

**Fig. 5:**
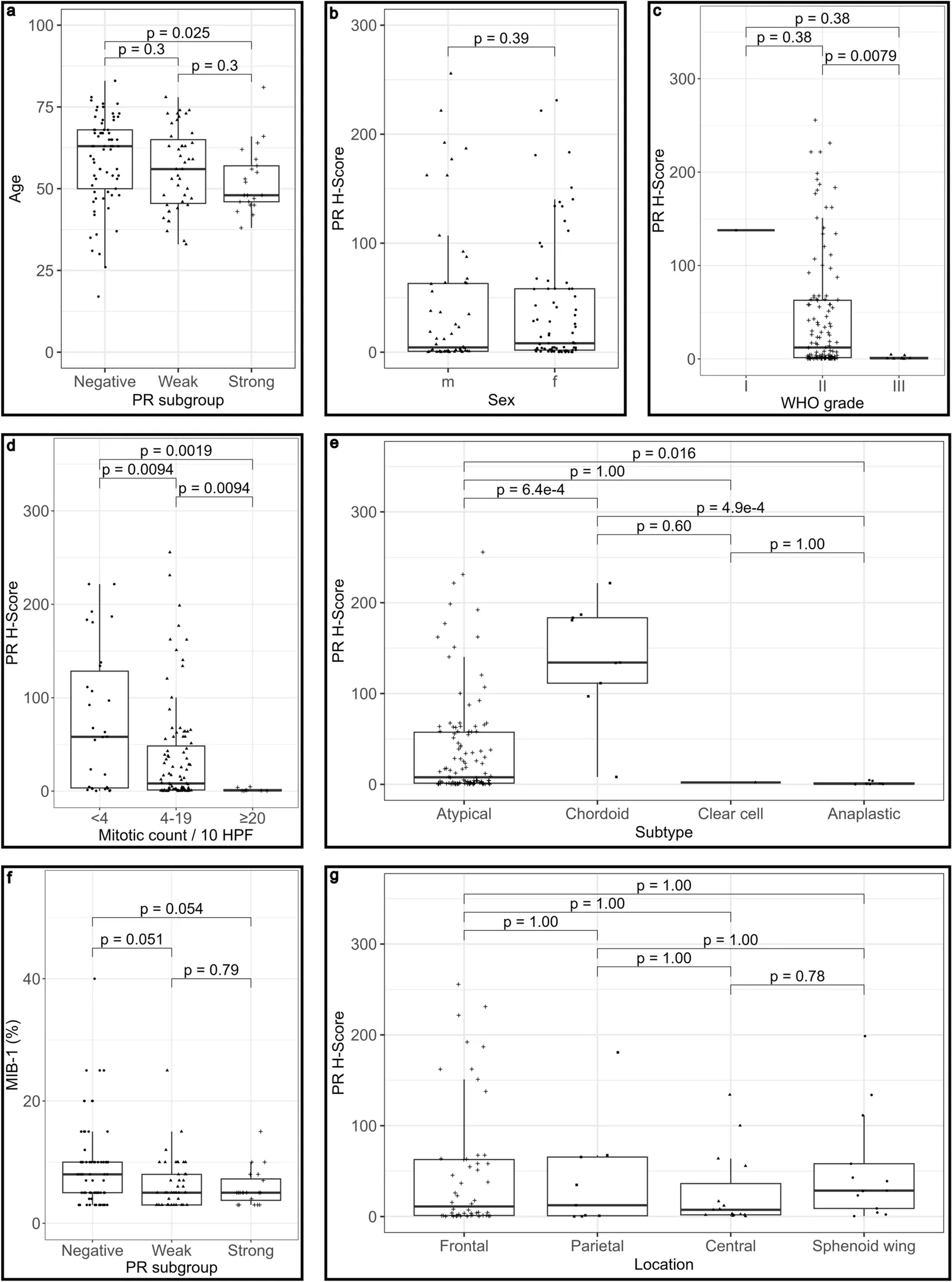
Clinicopathological parameters of the primary cohort in relation to PR expression of primary tumours. PR expression showed an inverse correlation with patient age, with significant difference between PR-negative and strong PR-positive tumours (**a**, *p*=0.025) and no association with patient sex (**b**). As indicated by proliferative activity (**f**), nuclear PR expression decreased significantly in tumours with higher mitotic activity (**d**). WHO grade was likewise inversely associated with PR expression (c, *p*=0.0079). Amongst histological subtypes, chordoid meningiomas exhibited the highest levels of nuclear PR expression (**e**). In contrast, neuroanatomical tumour location did not discriminate between PR expression categories (**g**)

Increasing PR expression was inversely correlated with grading, with WHO grade III meningiomas exhibiting significantly lower PR scores than WHO grade II tumours (*p*=0.0079; Fig. 5c), whilst chordoid meningiomas (WHO grade II) had the highest mean PR H-score (Fig. 5e). This association was further supported by an inverse correlation between PR expression and mitotic activity, as shown in Fig. 5d. Strong PR-positive meningiomas occurred more frequently in younger patients (*p*=0.025; Fig. 5a), whereas no differences were detected with respect to patient sex, MIB-1 index and location (Fig. 5b, f, g). Despite these associations, PR expression did not predict PFS in primary gross-totally resected WHO grade II meningiomas (Supplementary Fig. 4a).

### Influence of clinicopathological factors on prognosis of meningioma

**Fig. 6:**
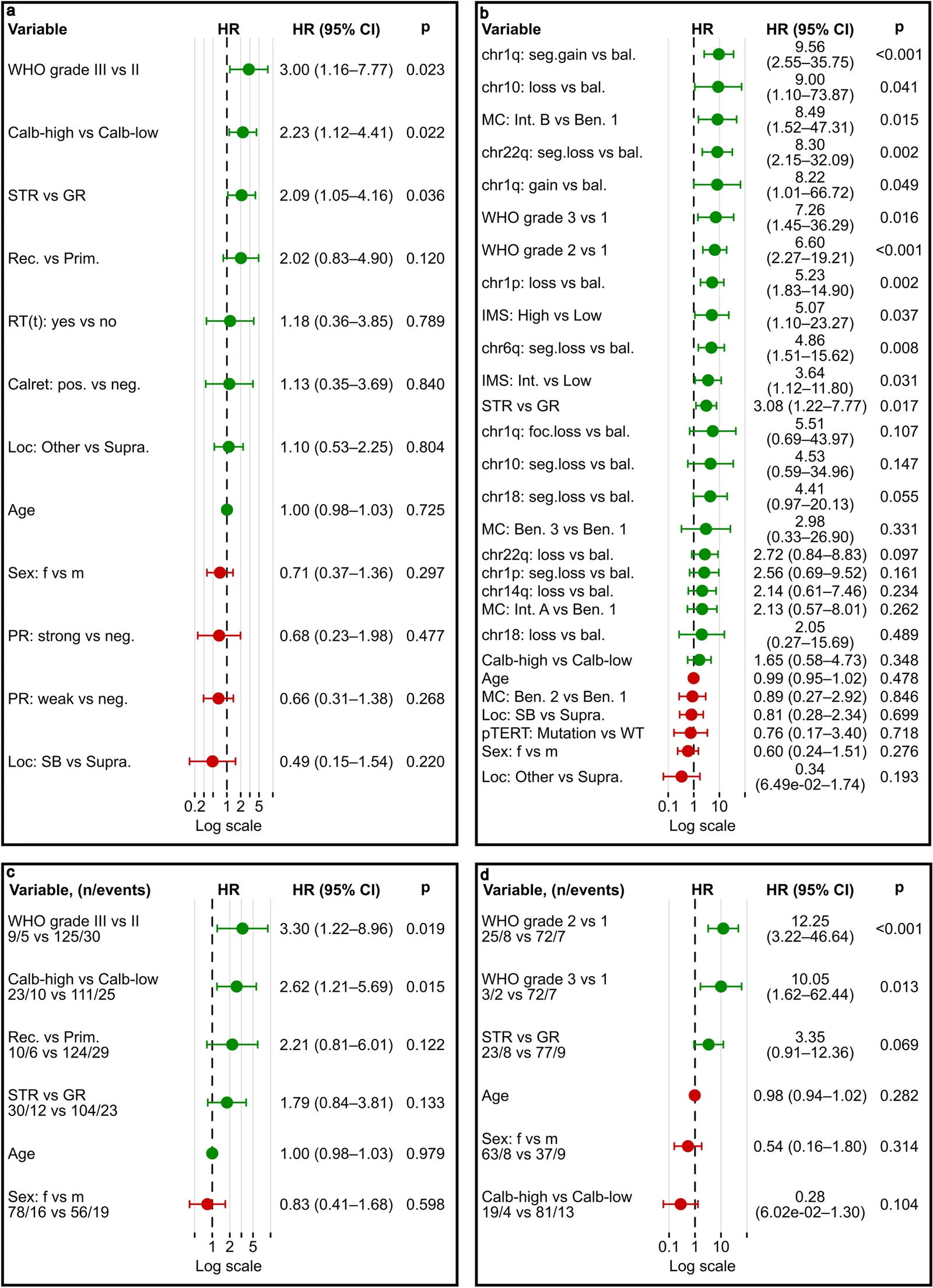
Associations of selected variables with PFS. **a**, **b** Univariable Cox analyses in the primary and secondary cohorts (RT(t), radiotherapy modelled as a time-dependent covariate). **c** The primary-cohort multivariable model included age, sex, primary versus recurrent disease status, extent of resection, Calb expression group and WHO grade. **d** The restricted secondary-cohort model included age, sex, extent of resection, CALB1 expression group and WHO grade. HRs with 95% confidence intervals and nominal *p* values are shown. Non-estimable coefficients were omitted from the plots. The limited number of progression events and absence of multiplicity correction should be considered when interpreting these exploratory analyses

The influence of Calb, Calr and PR expression, together with established clinicopathological parameters, on PFS was assessed using univariable and multivariable Cox proportional hazards models. In the primary cohort, most variables were not significantly associated with PFS, including age, sex, radiotherapy modelled as a time-dependent covariate, Calr and PR expression, tumour location and disease status. In contrast, a high Calb expression, subtotal resection and WHO grade III tumours were significantly associated with shorter PFS (Fig. 6a). In the primary cohort, a high Calb expression remained associated with shorter PFS after adjustment for age, sex, WHO grade, extent of resection, and primary versus recurrent disease status (HR 2.62, 95% CI 1.21–5.69; nominal *p*=0.015; Fig. 6c) in this cohort of predominantly high-grade meningiomas. Importantly, the association between a high Calb expression and a shorter PFS was also observed in analyses restricted to primary, gross-totally resected, non-irradiated, histologically WHO grade 2 meningiomas (Fig. 1c). This association persisted after further restricting the analysis to meningiomas classified exclusively by increased mitotic activity, thereby excluding chordoid and clear cell subtypes as well as tumours upgraded based on brain invasion or other non-mitotic criteria (*p*=0.011; Supplementary Fig. 4b).

In the secondary cohort, WHO grade, methylation subclass, several recurrent CNAs, IMS category, and extent of resection showed associations with PFS at nominal *p*<0.05 in exploratory single-variable analyses (Fig. 6b). As the restricted multivariable model included only 17 progression events, comprehensive multivariable survival modelling was not feasible without substantial risk of overfitting. In the restricted multivariable model, which included age, sex, extent of resection, modified integrated WHO grade, and *CALB1* expression group, a higher WHO grade remained associated with shorter PFS, whereas *CALB1* expression was not statistically significant (HR 0.28, 95% CI 0.06–1.30; nominal *p*=0.10; Fig. 6d). Likewise, Kaplan–Meier analysis restricted to primary, gross-totally resected WHO grade 1 meningiomas demonstrated no significant association between *CALB1* expression and PFS, although Calb-high tumours showed a numerical trend towards shorter PFS (*p*=0.17; Fig. 1f).

**Fig. 7:**
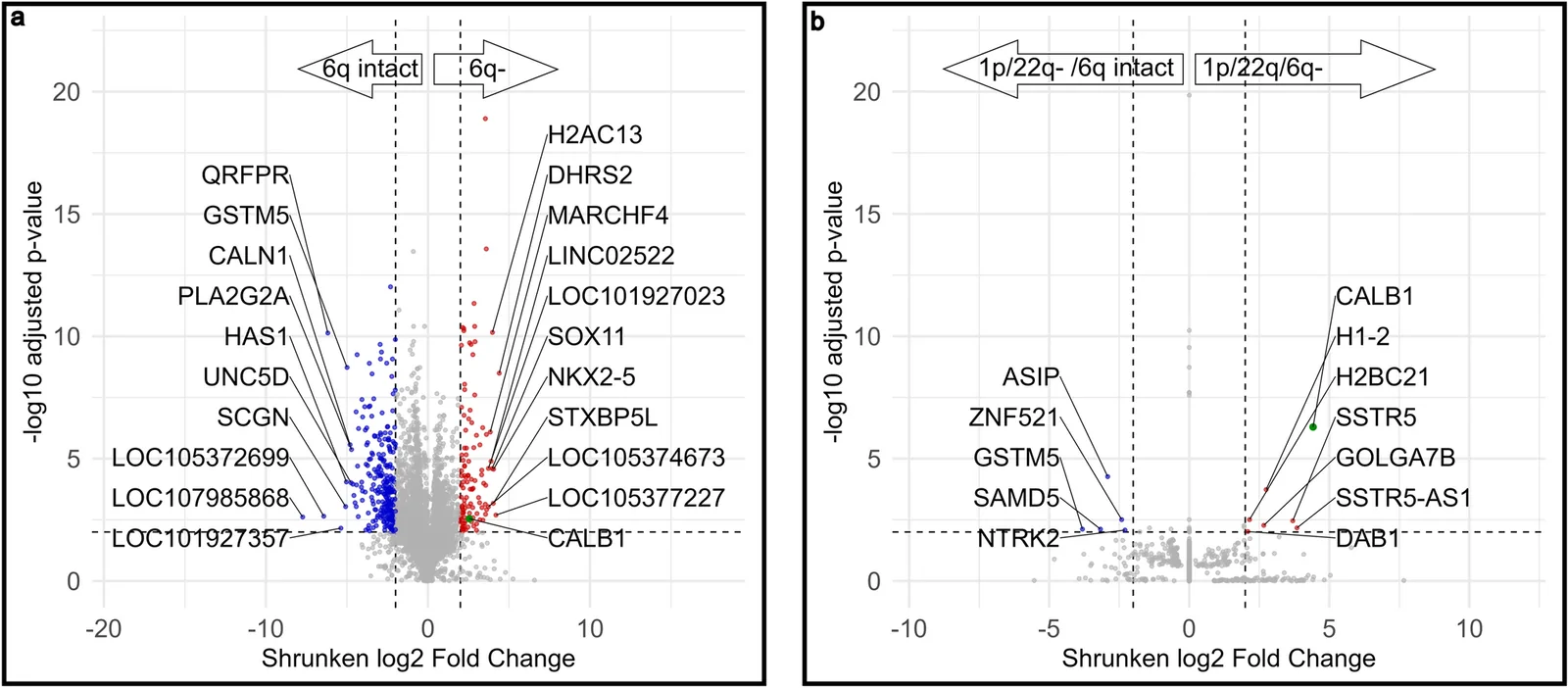
Gene expression differences associated with chromosome arm 6q loss in the secondary cohort. **a** Comparison of tumours with segmental or whole-arm 6q loss against all remaining eligible tumours. **b** Comparison restricted to tumours with combined 1p/22q loss, contrasting cases with and without additional segmental or whole-arm 6q loss. Cases with focal or mixed gain/loss alterations that precluded group assignment were excluded. The x-axis shows apeglm-shrunken log2 fold changes and the y-axis −log10 FDR-adjusted p values. Genes with FDR-adjusted *p*<0.01 and absolute shrunken log2 fold change >2 are highlighted in blue (lower expression) or red (higher expression) in the 6q-loss group; CALB1 is highlighted in green

As several recurrent chromosomal aberrations in meningioma are associated with adverse clinical outcome, identifying downstream transcriptional alterations may provide insights into the biological mechanisms underlying these molecular high-risk profiles. Chromosome arm 6q losses were identified in 11 of 110 tumours, including focal (*n*=1, 0.9%), segmental (*n*=8, 7.3%) and whole-arm losses (*n*=2, 1.8%; Supplementary File 2). The ten tumours with segmental or whole-arm 6q loss were included in the differential expression analyses; the tumour with focal 6q loss was excluded because it could not be assigned to either comparison group. A comparison of tumours harbouring segmental or whole-arm 6q loss with all remaining eligible tumours revealed differential expression of multiple genes, including *CALB1* and the transcription factors *NKX2-5* and *SOX11* (Fig. 7a). Within tumours with combined 1p/22q loss, *CALB1* showed the strongest upregulation and the lowest FDR-adjusted p value amongst upregulated genes in tumours with additional 6q loss (Fig. 7b). A chromosome-focussed multivariable linear regression model included sequencing batch and CNA categories for 1p, 1q, 6q, chromosome 10, 14q, chromosome 18 and 22q (*n*=95). In the focussed 6q-loss contrast, 6q loss remained associated with a higher *CALB1* transcript expression after adjustment for these covariates (nominal *p*=0.037; Supplementary File 1). In a separate clinical–molecular model including batch, 1p, 6q and 22q status, histological WHO grade, age, sex, *TERT* promoter status, methylation subclass and the *CALB1* CpG score (*n*=80), 6q loss was also associated with a higher *CALB1* expression (*p*=0.023; Supplementary File 1).

## Discussion

### Calbindin D28K expression and clinical outcome in meningioma

In the present study, we demonstrate Calb protein expression and elevated *CALB1* transcript levels in distinct subsets of meningiomas assessed in two independent cohorts. Approximately 30% of primary tumours showed detectable Calb immunoreactivity or elevated *CALB1* transcript levels, whereas the high-expression groups comprised approximately 18–19%. Normal meningeal cells showed absent or predominantly weak and sparse Calb immunoreactivity. In the primary cohort, a high Calb protein expression was associated with shorter PFS after adjustment for the included clinicopathological variables. This association was also observed in analyses restricted to histologically WHO grade 2 meningiomas. In contrast, *CALB1* transcript expression was not significantly associated with PFS in the restricted multivariable model of the secondary cohort. A numerical trend towards shorter PFS in its WHO grade 1 subgroup should be interpreted cautiously given the limited sample size and number of progression events. These findings support further investigation of Calb immunohistochemistry as a candidate adjunct marker for risk stratification, particularly within histologically WHO grade 2 meningiomas. Immunoreactivity was assessed using a modified H-score, providing a systematic semiquantitative assessment. Together with the predominantly diffuse staining pattern of Calb-high tumours, this may facilitate visual assessment and supports further evaluation of Calb immunohistochemistry as a rapid, potentially cost-effective adjunct marker for risk stratification in histologically WHO grade 2 meningiomas prior to molecular testing. Its reproducibility and incremental prognostic value require validation before clinical implementation.

Calb expression has previously been reported in several neoplastic entities, including medulloblastoma [17, 25], dysembryoplastic neuroepithelial tumour [34], subependymal giant cell astrocytoma [14, 21] and lung cancer [1, 7, 16]. Our findings extend these observations to meningioma and are consistent with the transcriptomic findings of Khan et al. [18], contrasting with earlier immunohistochemical observations in a small meningioma cohort [17]. Expression of the related calcium-binding protein Calr was rare and largely limited to scattered tumour cells, consistent with previous reports [31]. In the secondary cohort, multivariable clinical–molecular analysis identified associations of a higher *CALB1* expression with chromosome arm 6q loss, histological WHO grade 2, methylation subclass “Benign 3”, and lower methylation of *CALB1*-associated CpG sites, whereas methylation subclass “Intermediate B” showed an inverse association. Although chordoid meningiomas exhibited particularly a high Calb expression, the prognostic association persisted in a restricted subgroup excluding chordoid tumours, suggesting that it was not confined to this histological subtype. A possible relationship between Calb expression and a differentiation programme characteristic of chordoid meningiomas remains speculative. Together, these findings link *CALB1* expression to specific chromosomal and epigenetic features beyond conventional histopathological characteristics. Whether *CALB1* actively contributes to this programme or primarily represents a downstream marker remains unresolved.

Experimental studies in other cellular systems suggest that Calb can promote cellular survival by inhibiting caspase-3 activity [3, 5, 19], with both calcium- and pH-dependent mechanisms being proposed [5, 19]. In addition, Calb has been reported to reduce apoptosis through activation of the PI3K/AKT signalling pathway [33] and to facilitate escape from cellular senescence [1]. Notably, *CALB1* expression in the present study was not associated with increased mitotic activity or Ki-67 index, arguing against a predominantly proliferation-driven phenotype. Instead, previous studies have demonstrated reduced nuclear caspase-3 expression in an aggressive, drug-resistant subgroup of primary meningioma cell lines alongside transcriptomic evidence implicating *CALB1* overexpression [18], supporting the hypothesis that *CALB1* expression may promote tumour persistence through enhanced cellular survival and resistance to apoptosis rather than increased proliferation. Whether *CALB1* acts as a functional driver of these processes or represents a marker of this biological programme requires further investigation. The prognostic relevance of Calb expression appears to be highly context-dependent across tumour entities. A high expression has been associated with unfavourable outcomes in medulloblastoma [25] and in one report of non-small cell lung cancer [16], whereas variable or even favourable associations also have been reported in lung squamous cell carcinoma [1] and adenocarcinoma [7].

PR expression, an established differentiation marker in meningioma, was consistent with previous reports, showing an inverse correlation with WHO grade and generally favourable prognostic implications [27]. PR expression was not significantly associated with PFS in the univariable Cox and Kaplan–Meier analyses, consistent with previous studies reporting limited prognostic value beyond established histopathological parameters [28].

### Epigenetic regulation and potential upstream mechanisms

Our data support a possible contribution of DNA methylation to *CALB1* regulation. Hypomethylation of intronic and 1st exonic CpG sites correlated with increasing *CALB1* expression, indicating an epigenetic contribution to its regulation. Besides DNA methylation, *CALB1* regulation has been linked to ΔFosB, retinoic acid, vitamin D3, endogenous retroviruses and oestrogen signalling [1, 12, 35–37]. *ESR1* and *ESR2* are located on chromosome arms 6q and 14q, respectively. In our analyses, 6q loss was associated with a lower *ESR2* expression but not with significantly altered *ESR1* expression. Loss of 14q was associated with a lower *ESR2* and a higher *ESR1* expression. However, neither receptor transcript showed a significant association with *CALB1* expression in batch-adjusted linear or rank-based analyses. These findings do not support a simple relationship between oestrogen receptor transcript abundance and *CALB1* upregulation.

In the clinical–molecular multivariable model, chromosome arm 6q loss remained associated with a higher *CALB1* expression after adjustment for the included clinicopathological variables, methylation subclass, selected CNAs, and a methylation score derived from the *CALB1*-associated CpG sites cg26290632, cg12402251, and cg18335796. A separate chromosome-focussed model also supported this association after adjustment for additional recurrent CNAs. Together, these findings associate *CALB1* expression in meningioma with specific epigenetic and chromosomal features. The molecular mechanisms underlying these associations remain unresolved. Functional studies are required to determine whether *CALB1* contributes to the associated biological phenotype or primarily represents a downstream marker.

### Limitations and clinical implications

This study has several limitations. Intra-tumoural heterogeneity may introduce sampling bias in tissue microarrays. In the secondary cohort, *CALB1* transcript expression, DNA methylation, and CNA status were assessed in the same tumours, enabling direct transcript-level analyses of their associations. However, Calb immunohistochemistry was performed in an independent cohort. The absence of integrated protein, transcript, and CNA assessment therefore precludes direct validation of the relationship between chromosome arm 6q loss and Calb protein expression. Future studies assessing all three levels in the same tumours are required. The association between 6q loss and *CALB1* transcript expression persisted after adjustment for recurrent CNAs and in analyses restricted to tumours with 1p/22q codeletion. However, the co-occurrence of these alterations and the limited number of 6q-loss tumours constrain conclusions regarding specificity for 6q loss.

The limited number of progression events in the secondary cohort also constrained multivariable survival modelling and increased the risk of overfitting. The present study therefore does not establish incremental prognostic value beyond established clinicopathological and molecular risk factors. Finally, functional studies are needed to determine whether *CALB1* contributes to tumour progression or represents a marker of the associated molecular programmes.

Despite these limitations, a high Calb protein expression was associated with shorter PFS in the primary cohort after adjustment for the included clinicopathological variables, with supporting findings in analyses restricted to histologically WHO grade 2 meningiomas. Complementary same-sample molecular analyses in the secondary cohort linked *CALB1* transcript expression to chromosome arm 6q loss and specific epigenetic features. Together, these findings support further evaluation of Calb immunohistochemistry as a candidate adjunct marker for risk stratification, particularly in histologically WHO grade 2 meningiomas. The molecular findings also provide a rationale for investigating a possible relationship between *CALB1* regulation and cellular survival in meningioma, without establishing a causal mechanism.

## Supplementary Information

Below is the link to the electronic supplementary material.

Supplementary file1 (PDF 906 KB)

Supplementary file2 (XLSX 23 KB)

Supplementary file3 (XLSX 11 KB)

## Data Availability

All data supporting the findings of this study are included in the article and its supplementary information or are available from the corresponding author upon reasonable request. Source data are provided with this publication.
