# Supplementary material for: CALB1 expression in meningioma: associations with progression-free survival and chromosome arm 6q loss
Source: Acta Neuropathol. 2026 Nov 5;152(1):66. doi: 10.1007/s00401-026-03113-3 (PMC13739945; doi:10.1007/s00401-026-03113-3)
Supplement: Supplementary file 1 [file 401_2026_3113_MOESM1_ESM.pdf]

***CALB1* expression in meningioma: associations with progression-free survival and chromosome arm 6q loss – Supplementary Figures**

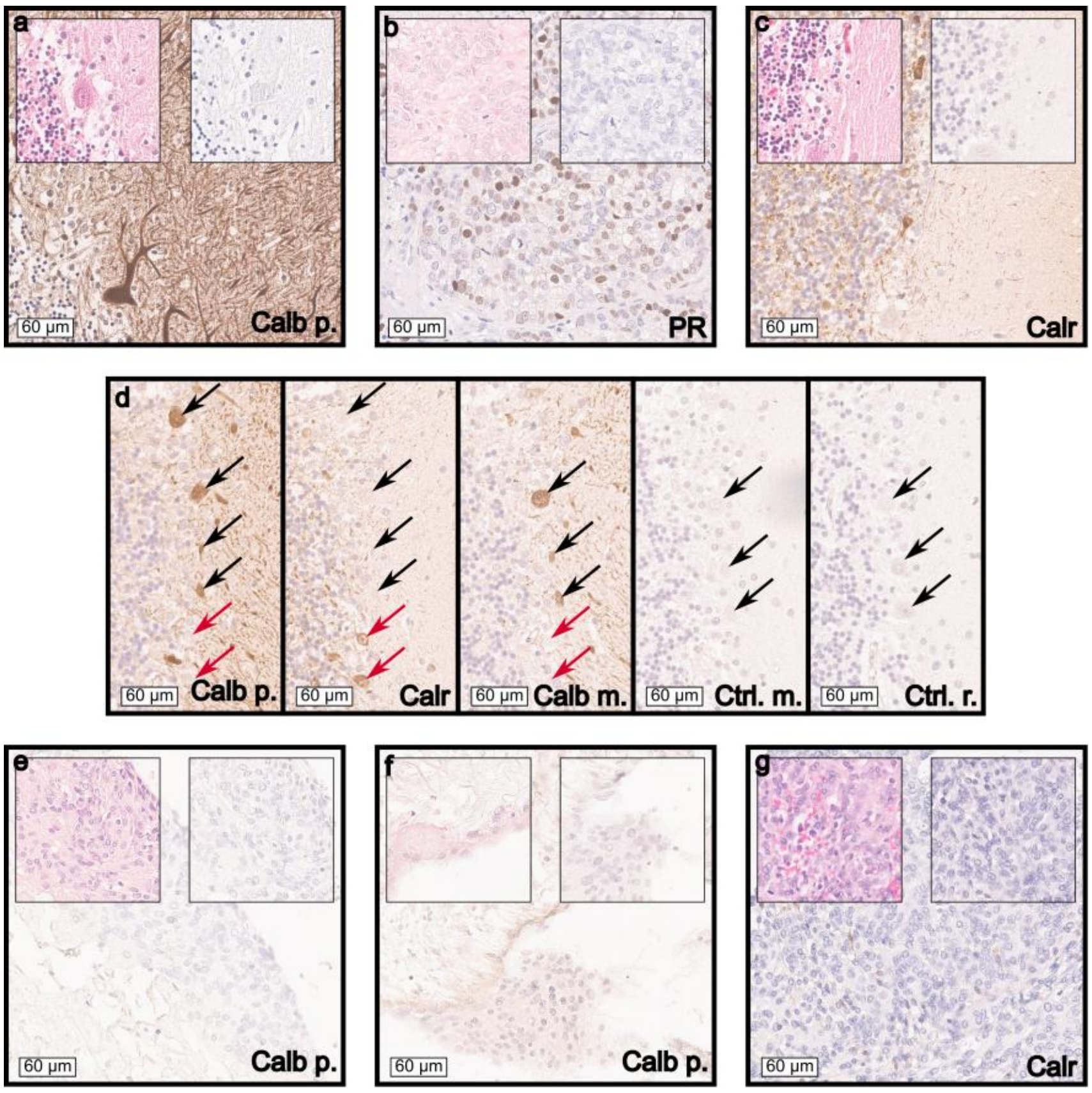

Supplementary Figure 1 Immunostainings. (a-c) Positive control stainings showing human cerebellum for Calb (a) and Calr (c), as well as breast carcinoma metastasis for PR (b). Insets: corresponding H&E staining (upper left) and negative control with omission of the primary antibody (upper right). (d) Serial sections of human cerebellum demonstrate specific immunoreactivity of both the monoclonal (C9848) and polyclonal (CB38a) anti-Calbindin antibodies in Calb expressing Purkinje neurons (black arrows). Conversely, Calr expressing unipolar brush cells (red arrows) display immunoreactivity exclusively with the anti-Calretinin antibody. From left to right: polyclonal anti-Calbindin (CB38a), polyclonal anti-Calretinin (CR7697), monoclonal anti-Calbindin (C9848), negative control with omission of the primary antibody (mouse) and negative control with omission of the primary antibody (rabbit). (e, f) Immunostainings of meningeal samples demonstrating predominantly no Calb expression (e) or weak staining in basal layers of arachnoid cap cells (f). (g) Example of singular scattered Calr-positive meningioma cells in an anaplastic meningioma, WHO grade III

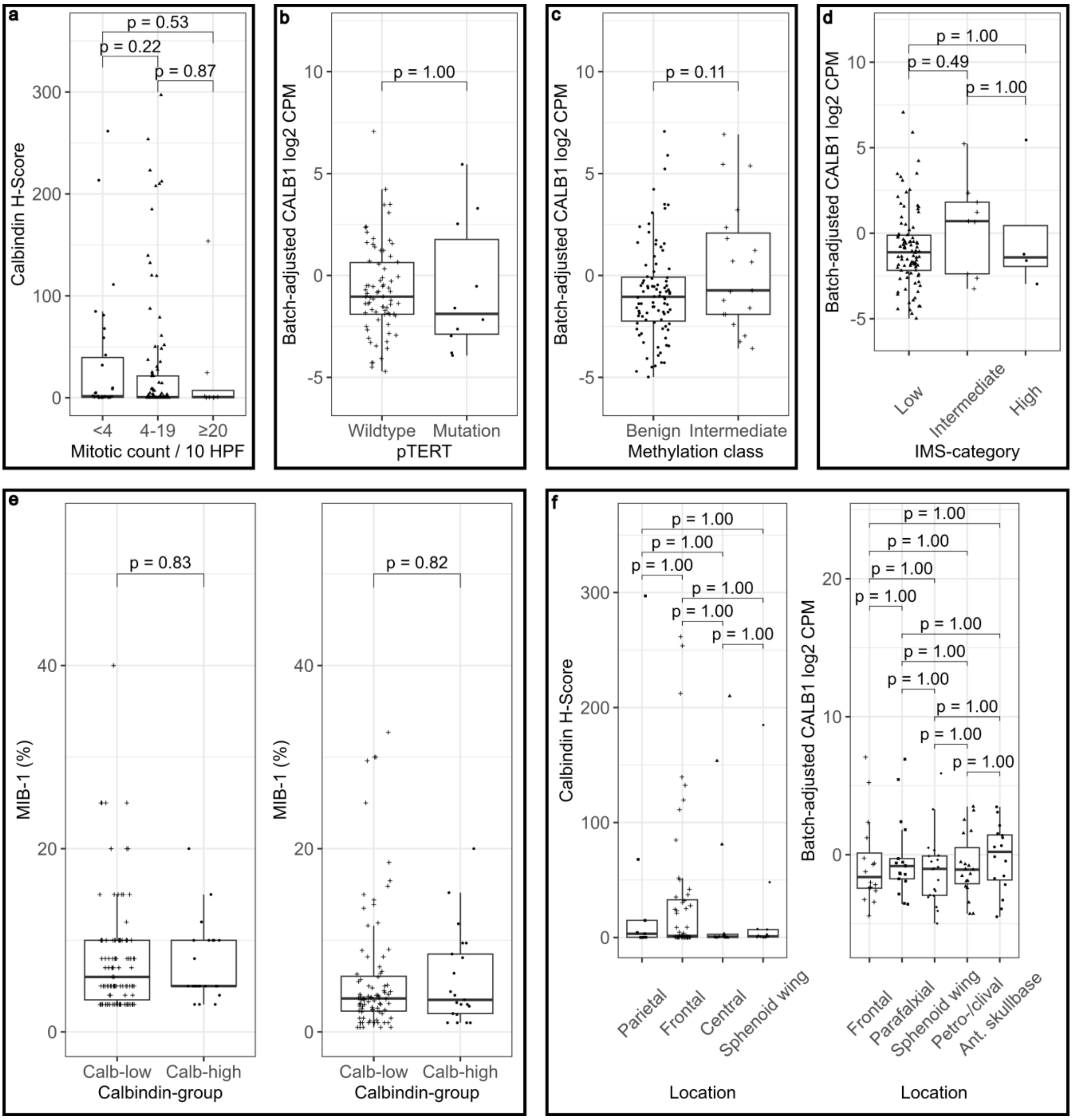

Supplementary Figure 2 Clinicopathological parameters of the primary and secondary cohort in relation to Calb expression. Mitotic count (a) and proliferative activity (e) showed no significant association with Calb expression in either cohort. Likewise, neuroanatomical tumour location (f), *TERT*-promoter mutation status (b), methylation class (c) and IMS category (d) failed to discriminate between different Calb expression levels in the corresponding group comparisons

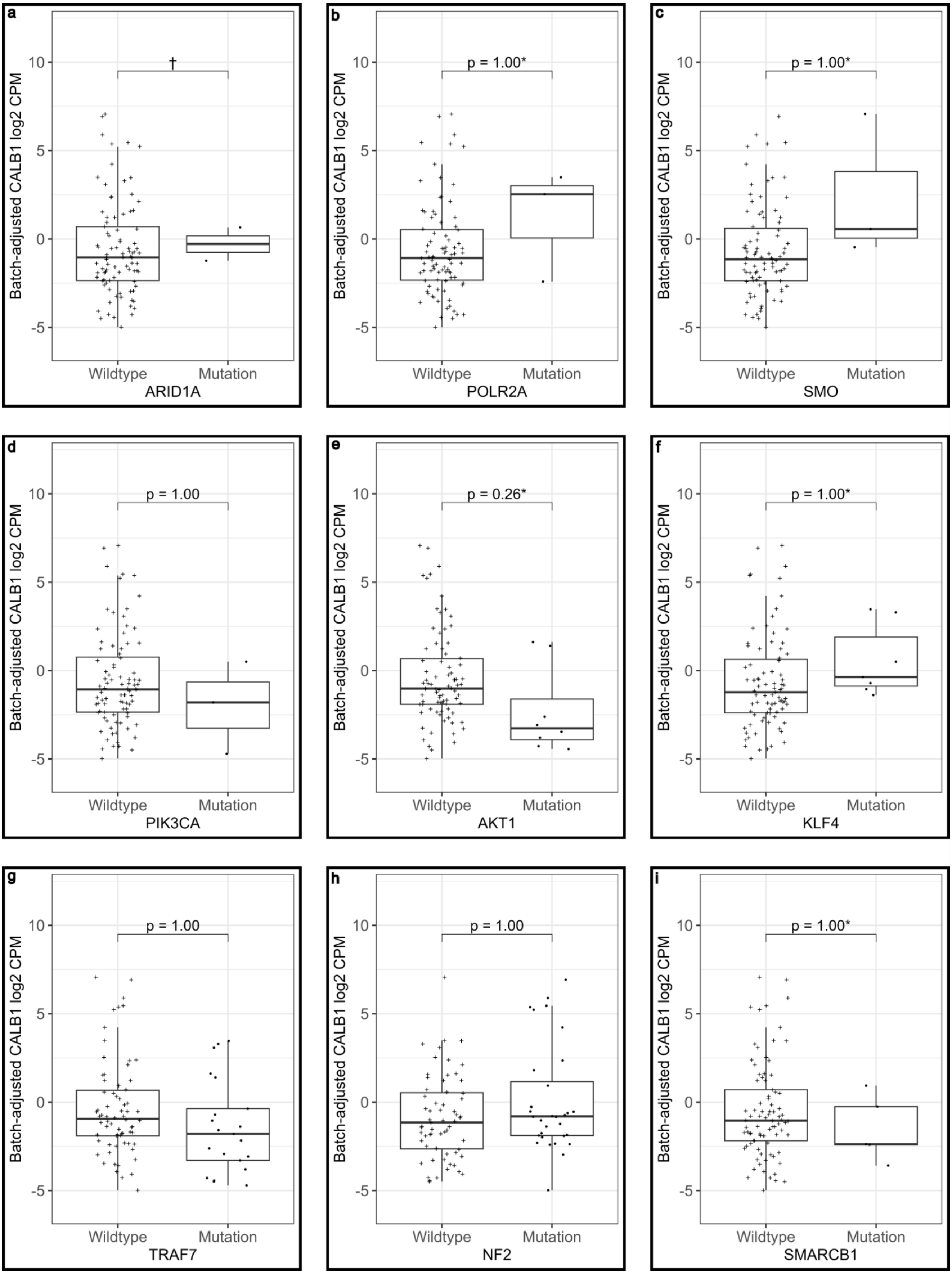

Supplementary Figure 3 *CALB1* transcript expression by recurrent driver-mutation status in the secondary cohort. No evaluable comparison retained statistical significance after adjustment for multiple testing. Asterisks (*) identify comparisons calculated within a single sequencing batch. †*ARID1A* was displayed descriptively but excluded from inferential analysis because only one mutant tumour was available within the batch containing both mutation-status groups

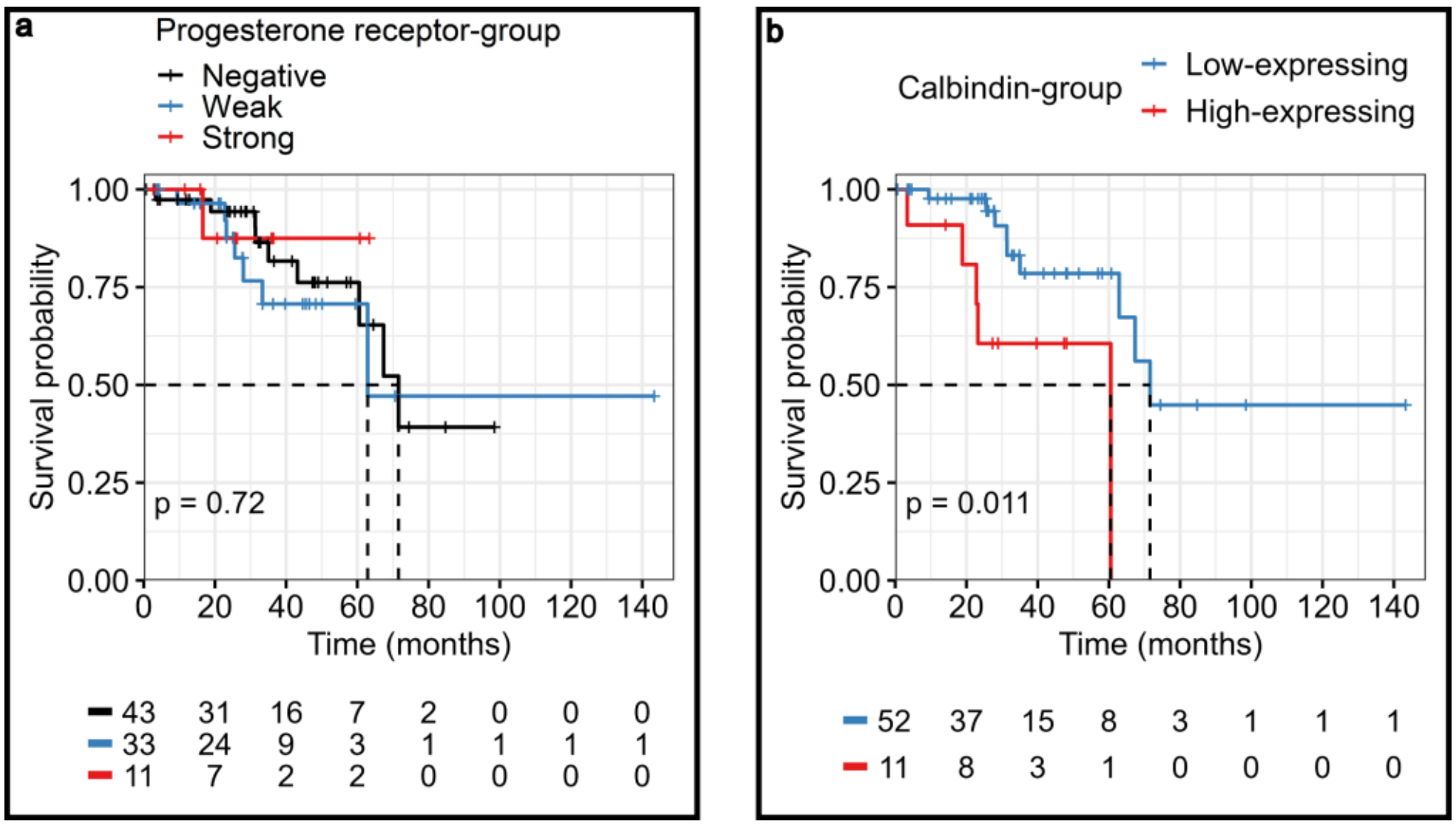

Supplementary Figure 4 PFS of PR- and Calb-groups (primary cohort). Negative, weak and strong PR expressing primary, gross-totally resected WHO grade II meningiomas without radiotherapy did not show any significant differences of progression-free survival (a). (b) High Calb-expression of primary, gross-totally resected, non-irradiated WHO grade II meningiomas classified exclusively by increased mitotic activity remained significantly associated with worse prognosis after exclusion of chordoid and clear cell morphology, as well as brain invasion and "soft criteria" as grading features (p=0.011)
